# Molecular docking analysis of C-phycocyanin with VEGFR2

**DOI:** 10.6026/97320630016869

**Published:** 2020-11-30

**Authors:** Saira M Bannu, Dakshayani Lomada, Sravani Varala, Madhava C Reddy

**Affiliations:** 1 Department of Biotechnology and Bioinformatics, Yogi Vemana University, Kadapa AP 516005, India; 2 Department of Genetics and Genomics, Yogi Vemana University, Kadapa, AP 516005, India

**Keywords:** Molecular docking, C-Phycocyanin, VEGFR2, Angiogenesis, Cancer treatment

## Abstract

C-phycocyanin (C-PC) produced from cyanobacterial species finds application in drug development. Therefore, it is of interest to document the molecular binding features of C-PC with the vascular endothelial growth factor receptor 2 (VEGFR2). C-PC showed H-bond interactions with residues on both sides of the Deusche Forschugsgemein-Schalt (DFG) loop (Asp1046-Phe1047-Gly1048). A hydrophobic association between the activation loop and the DFG residue (Gly1048) helps to inhibit the activity of VEGFR2 kinases. Thus, C-PC is reported as a potential angiogenesis inhibitor for VEGFR2 in combating cancer.

## Background

C-Phycocyanin (C-PC) is a light yielding protein pigment produced from Cyanobacterial species. We recently reviewed therapeutic applications of C-PC [1]. C-PC shown to have anti-cancer properties [2,3] immunity promoting effects [4], antioxidative [5], anti-inflammation [6], and Neuro protective [7]. C-PC also shown to be nontoxic photo-sensitizer utilized in the adjuvant photodynamic therapy (PDT) of tumors [8]. Angiogenesis was the procedure of formation of new blood vessels by regulating the chemical signals such as VEGF, which binds with the receptors on the surface of endothelial cells. VEGF receptors are classified into three types: VEGFR1, VEGFR2 and VEGFR3 respectively [9]. VEGFR2, expressed mostly in endothelial neovascular tumor cells, is generally identified in endothelial vascular cells by a gene encoding the kinase insert domain receptor (KDR) [10]. The availability of data on molecular docking studies, dynamics simulations and bioinformatics tools has provided ample scope for identifying novel inhibitors against human VEGFR2, the molecular target of the Swiss army knife in lung cancer [11]. Earlier we showed that C-PC acts as a Cyclooxygenase -2 (COX-2) inhibitor and COX-2 inhibitors are potent angiogenesis inhibitor [12,13]. Therefore, it is of interest to document the molecular binding features of C-PC with the vascular endothelial growth factor receptor 2 (VEGFR2).

## Materials and Methods:

VEGFR2 (target) is docked with C-PC by using Auto Dock 4.2. The dynamics analysis was done using Desmond V3.2 (Table 1 - see PDF) [14]. PDB is a structural database for protein. The crystal structure of VEGFR2 was retrieved from the Protein Data Bank (PDB), and C-PC was used as a ligand for docking simulation studies using Auto Dock 4.2 and Desmond V3.2. Protein was prepared by adding Hydrogens, polar groups and non-polar groups for boundering the membrane conditions for the protein, while in the case of ligand preparation the same steps are followed by the addition of Kolman charges to the respective ligand. These files were saved in PDBQT format. Grid was generated (60x60x60) xyz points, grid spacing was 0.375 Å and center of grid dimensions were 1.095, -1.554 and 3.894. The score of grid was determined to decline the computation time in contrast to the ligand structure. Auto Dock's primary aim is to dock protein ligand information along with the grid box properties in the configuration file, and also engaged in global local search optimizer whereas the ligand and the protein were arbitrated as rigid. The result in RMSD was less than 1.0Å was described as most free energy of binding. For future analysis the lowest binding or binding affinity was extricated and aligned with the receptor structure [11,15].

## Docking studies:

Complications such as loops, missing hydrogen atoms with flipped residues. The incomplete side chains were enhanced using Auto Dock. Energy minimization, refinement and minimization of the protein were performed in this study. Affinity maps (grid) with a spacing of 0.375 Å were generated using Auto grid program around the receptor-ligand complex binding site [16]. The parameters of Auto Dock functions such as di-electric set and distance dependent were used in the computation of electrostatic and Vander Waals respectively. An orientation, initial position, and torsions have been set at random. After the docking, all the rotable torsions were released which were acquired from 10 separate runs that were scheduled to end after a maximum energy assessment of 2,50,000 [17]. The size of the population was set to 150 Å translational step of 2.0Å, quaternion and 5 torsion steps were applied [18]. Further, the binding orientations of C-PC with VEGFR2 were predicted through Molecular Dynamics (MD) simulations.

## Molecular Dynamics analysis:

The stability of the VEGFR2 and C-PC docked complex was crosschecked using the force field Optimized Potentials for Liquid Simulations (OPLS)-2005 via the 100ns MD simulation at Desmond (v3.8). Desmond was unified with molecular modeling to simulate chemical and biological systems, which compares with widely used analytical and trajectory viewing tools. The Desmond based simulations follow the systematic workflow for system building with the six steps relaxation protocol followed by MD simulations. The system builder allows building a solvated system for protein-ligand complex by choosing parameters for water model, force field, ensemble type, thermostats, barostats algorithms, adding ions, salt and defining periodic boundary conditions, etc. The six steps equilibration process was performed as described (1) Minimization of the solvated system with solute was restrained by hybrid algorithm method of steepest descent and limited memory Broyden Fletcher Goldfarb Shanno (LBFGS) (2) Minimization without solute restraints by the steepest descent and LBFGS algorithms (3) minimization of system for 12 ps simulation with NVT ensemble at temperature of 10K with restrained non-hydrogen solute atoms, (4) Again system was minimized in the NPT ensemble, 12 ps at 10K (5) System minimization was carried out for 24 ps simulations in NPT ensemble temperature of 300K restraining the non-hydrogen solute atoms. (6) At temperature 300K no restraints in NPT ensemble at 24 ps simulation were used [19]. MD simulation through the solvated system was built by fixing the Simple Point Charge (SPC) model for the characterization of water molecules over the docked complex at 300k temperature by neutralizing the system with Na+ and Cl- ions for balancing the net charge to neutral the whole simulation box. OPLS-AA 2005 was used by the protein-ligand systems for the force field parameters. For the relaxed system simulation with 50ns time with 2fs isothermal isobaric ensemble (NPT) all at once using Nose Hoover Thermostat at 300k, 1,01325 bar pressure with Martyna-Tobias-Klein Barostat system energies and atomic data coordination data was listed for every 1.2 and 4.8 ps [18]. RMSD, RMSF, ligand-protein interactions and radius of gyration (r Gyr) of the VEGFR2-C-PC system were analyzed in 10,416 trajectories.

## Results and Discussion:

### Molecular docking:

**Figure 1:**
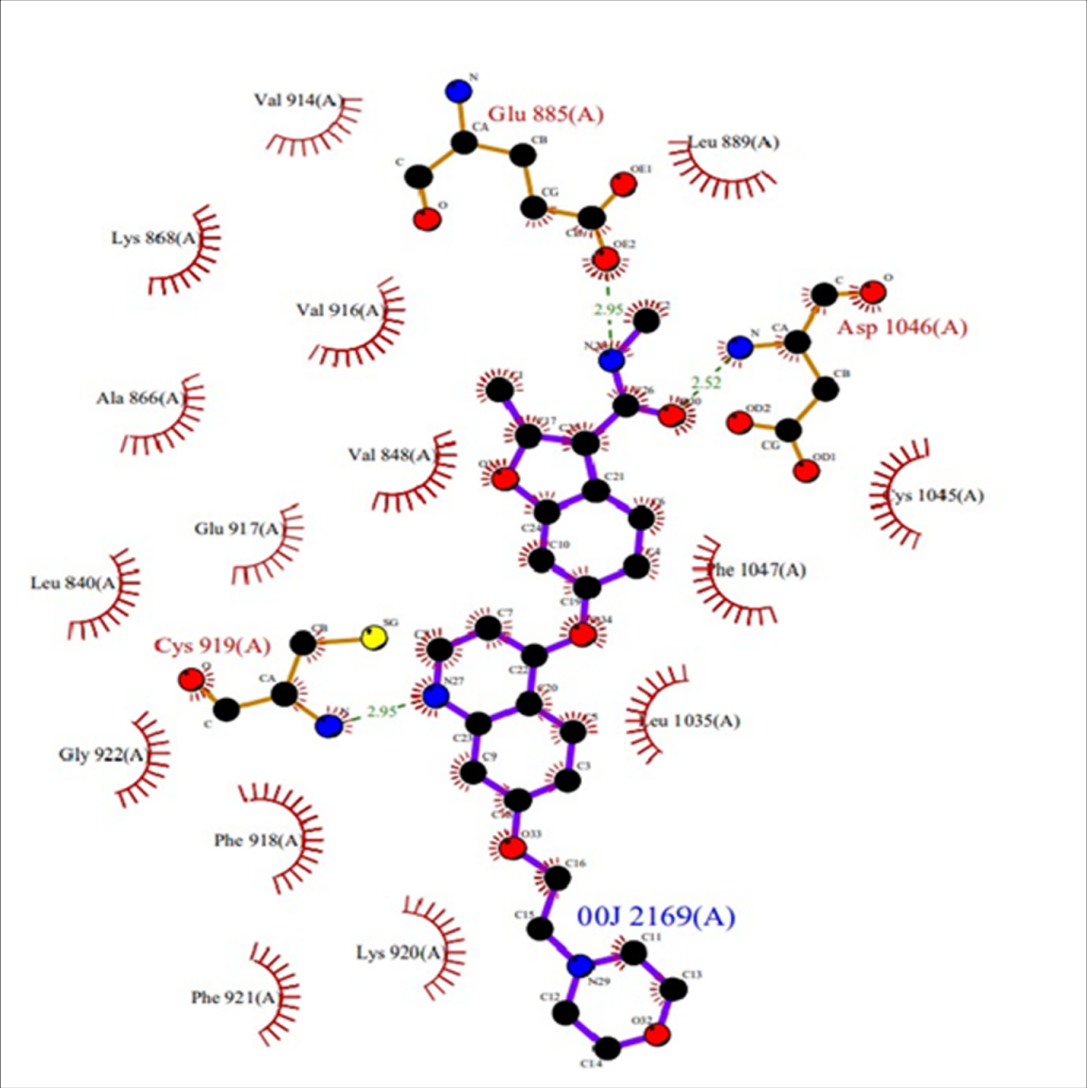
Binding site residues of VEGFR2 (PDB ID: 2XIR) with co-crystal ligand

**Figure 2:**
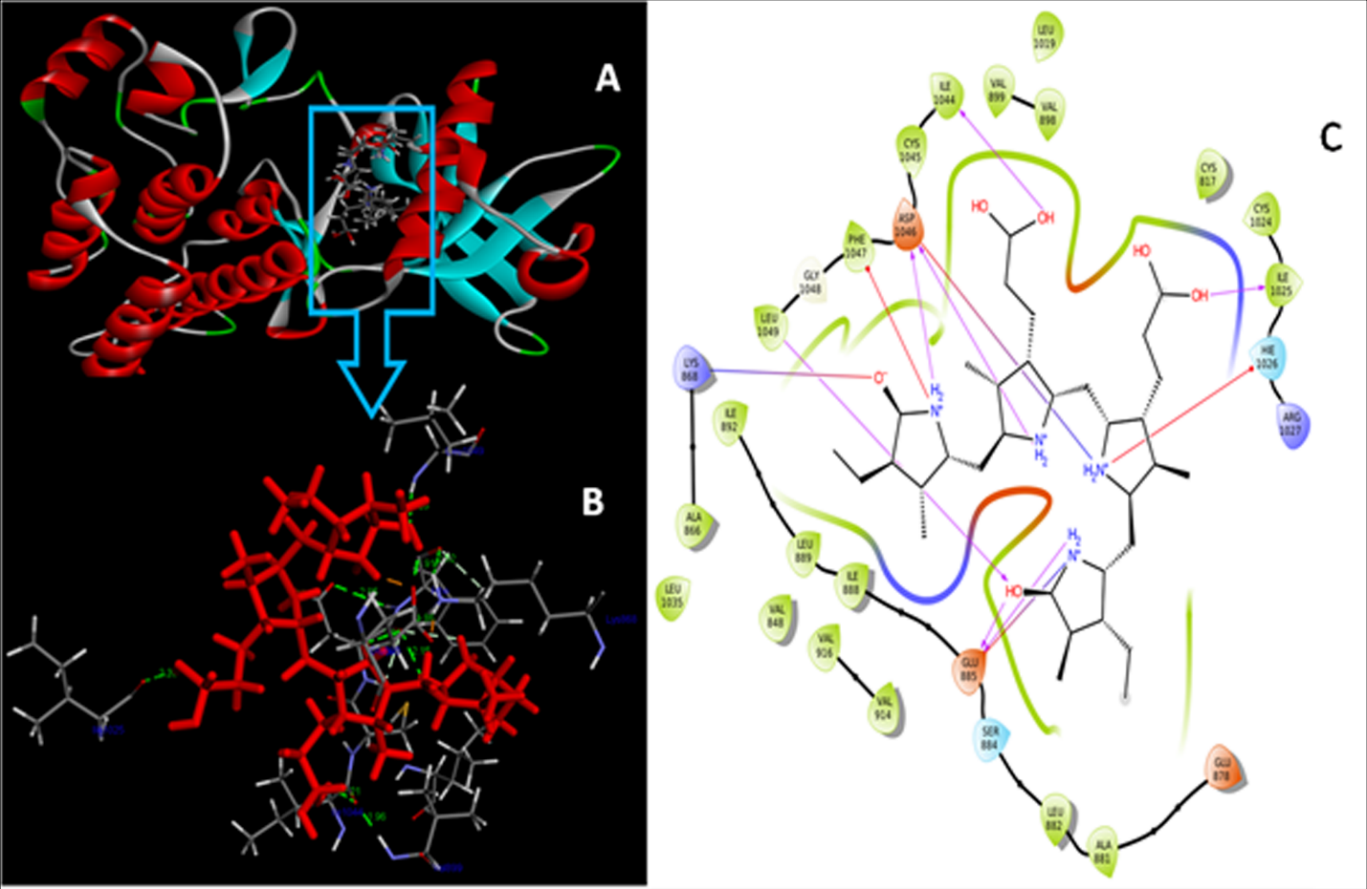
(A) Docking interaction of VEGFR2 and C-PC; (B) Binding interaction of protein with the ligand; (C) Amino acid interactions of C-PC and VEGFR2

Considering the VEGFR2 as valid target for docking studies revealed that C-PC was binding accurately by forming the well-established hydrogen bonds with the active site residues. Grid was formed around the co-crystal ligand (N, 2-dimethyl-6-[7-(2-morpholin-4-ylethoxy) quinolin-4-yl]oxy-1-benzofuran-3-carboxamide) binding site residues Leu840, Val848, Glu885, LYS868, Val916, Leu889, Val914, Cys919, Phe918, Glu917, Lys920, Phe921, Gly922, Leu1035, Cys1045, Asp1046, Phe1047 (Figure 1). The docking studies revealed that C-PC docked with extreme residues that constituted the major binding sites (active site). Analysis of our results were evaluated that C-PC with VEGFR2 showed better binding interactions when compare with co-crystal ligand. C-PC showed the binding energy of -10 kcal/mol; inhibition constant of 46.88 nM. C-PC formed four single hydrogen bond interactions with Glu885 (α-C glutamate residue), Ile1025, Leu1049, Ile1044 and double hydrogen bond interaction with Asp1046 (Second D of K/D/D loop) (Figure 2). Additionally salt bridge interactions were observed with Lys868 (K of K/D/D loop), Glu885, Asp1046 and one Pi-interaction were formed with Phe1047. C-PC interaction with the Glu885 (α-C glutamate residue) will help to break down the salt-bridge between β3-lysine and C-glutamate, which results in the inactive form of VEGFR2 conformation in cancer conditions. According to Hanks and Colleagues Lys868 shows catalytic property of VEGFR2. The C-PC forming hydrogen bond with the Lys68 which in turn ion pair breakage will takes place between the α and β phosphates of ATP with Glu885 of the α-C helix. Asp1046 is the active site residue of the active site loop in a conserved DFG (Asp-Phe-Gly) loop. Binding of C-PC with the Asp1046, which inhibit the Mg2+ binding mechanism, which in turn no bond formation between the α and β phosphate groups of ATP. By all the mechanistic observations of the VEGFR2, the C-PC is binding more accurate in active site regions which helps to inhibit the over expression levels of protein in cancers. Further docked complex interactions and stability was evaluated through 100ns molecular dynamics simulations.

### VEGFR2- C-PC complex dynamics analysis:

**Figure 3:**
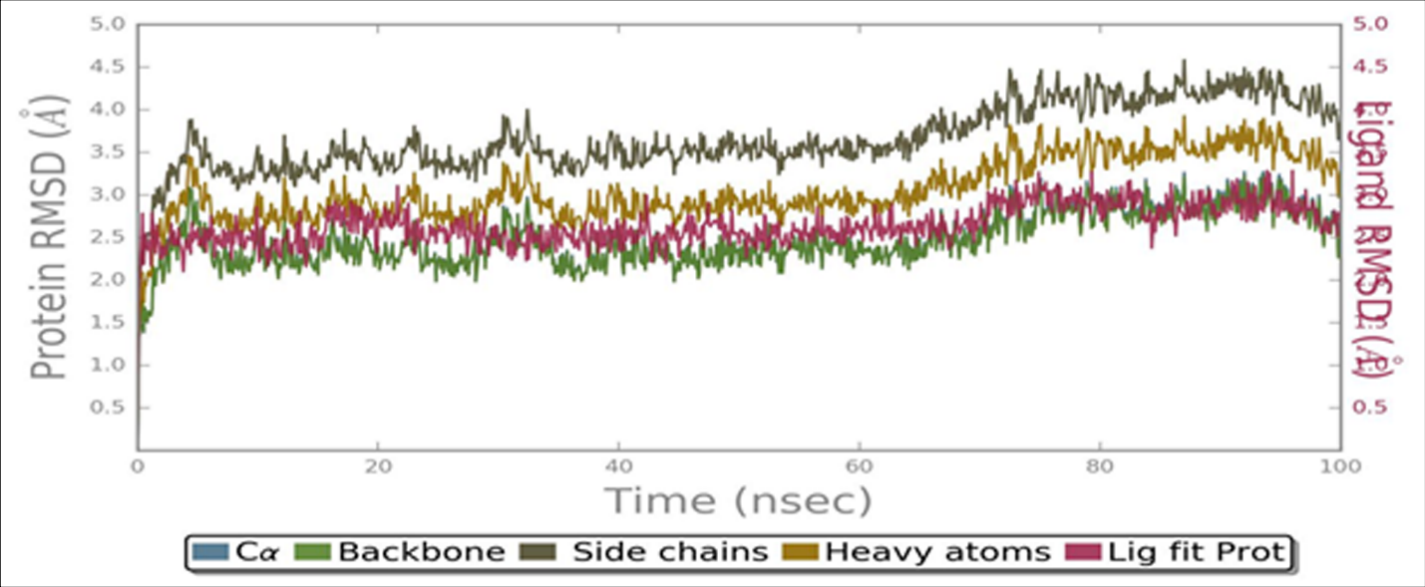
RMSD of VEGFR2-C-PC complex against the distance in Å (Y-axis) and number of trajectories (X-axis)

**Figure 4:**
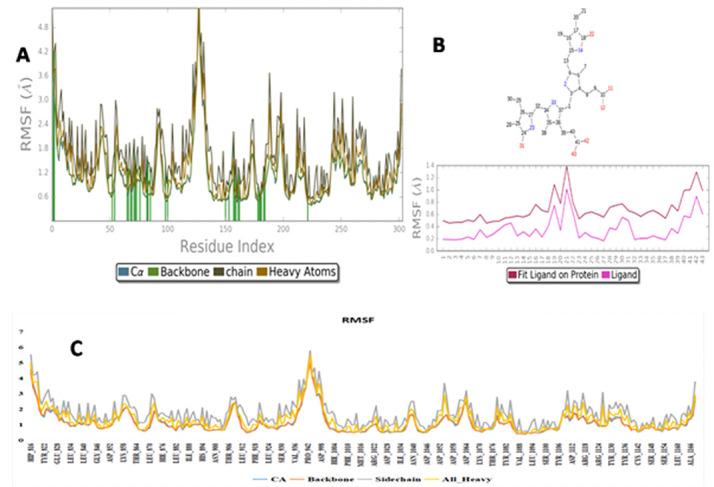
(A) Average RMSF of C-alpha, Backbone, Side chain and Heavy atoms; (B)Protein and Ligand fit (RMSF Interactions); (C) RMSF of VEGFR2-C-PC complex over the simulation period against distance in Å (Y-axis) and residues (X-axis)

Stability and detailed interactions between the C-PC with VEGFR2 were explored. Total energy, RMSD, RMSF, ligand binding interactions and gyration energy was analyzed in 10416 trajectories. Total energy of the system was observed of -161100 kcal/mol. Average RMSD was observed for back bone is 2.4 Å; C-alpha is 2.4Å; Side chain 3.6 Å; heavy atoms is 3 Å; ligand with protein is 2.6 Å and ligand with ligand was observed below 1.4 Å. RMSD reveals that back bone and C-alpha was observed below the 3Å (Figure 3). Where as little fluctuations were observed in side chain region but it maintained stability throughout the simulations period. Ligand was maintained its stability at 1.5 Å and showed good binding stability with the VEGFR2 protein.Local fluctuations of VEGFR2-C-PC docked complex were calculated by the RMSF analysis. Initially, till 50 trajectories RMSF was reached nearly 6 Å later on RMSF of the protein was maintaining the steady state below the 3 Å (Figure 4). From 325 - 375 trajectories there was little more fluctuations was observed Val936, pro942 and Asp998. But it is not effecting the structural conformation of the complex because active site residues were maintaining the steady nature with the acceptable range RMSF under the 3Å. Average RMSF of C-alpha, Backbone, Side-chain and Heavy atoms were observed like 1.1 Å, 1.1 Å, 1.7 Å and 1.3 Å. Overall, RMSF analysis revealed that the VEGFR2 maintained its stability throughout the 100 ns simulations with very lesser fluctuations it gives the binding affinity towards the C-PC molecule.

**Figure 5:**
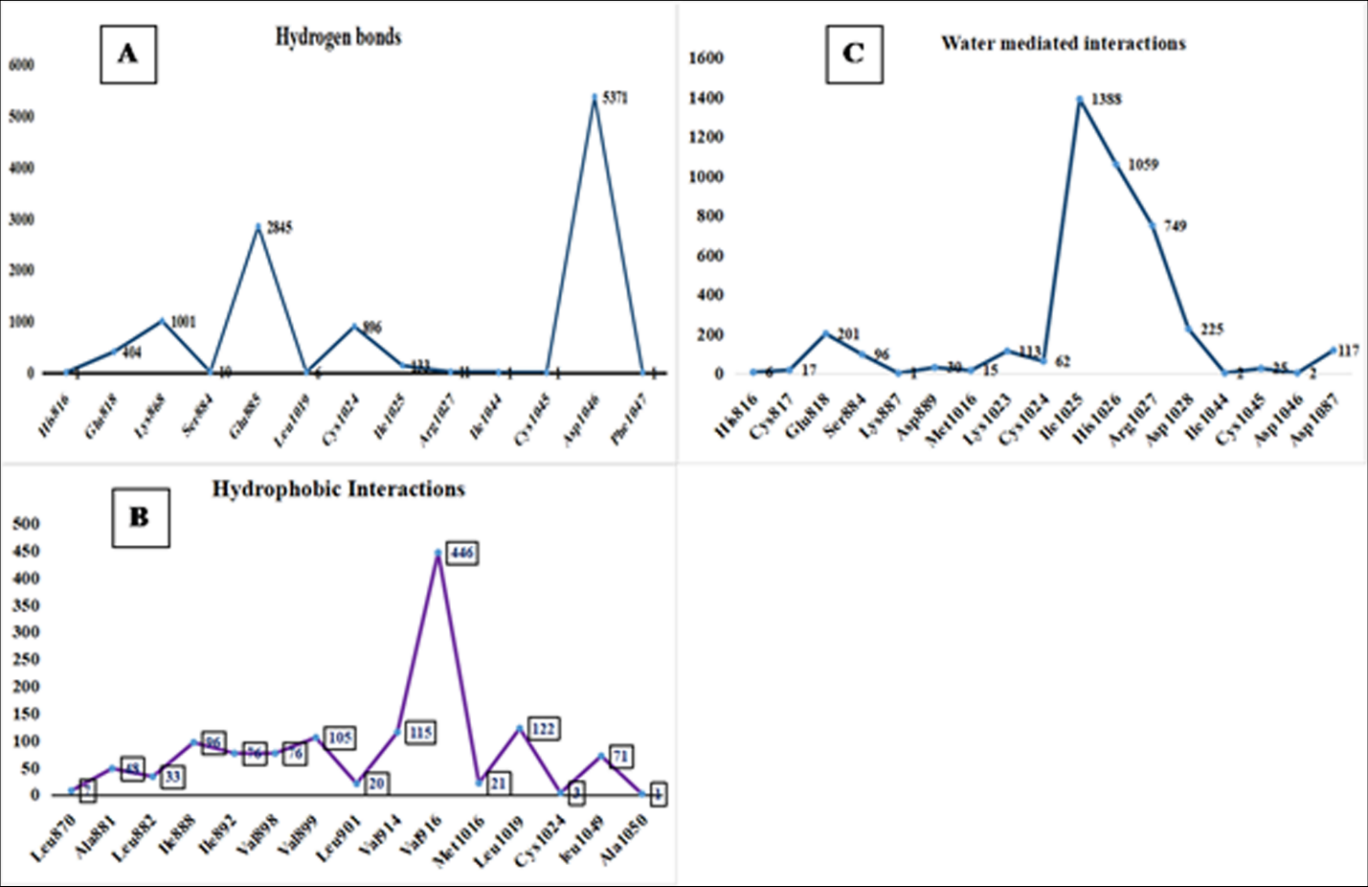
VEGFR2- C-PC docked complex residue interactions against trajectories (A) Hydrogen bond (B) Hydrophobic and (C) Water mediated interactions

**Figure 6:**
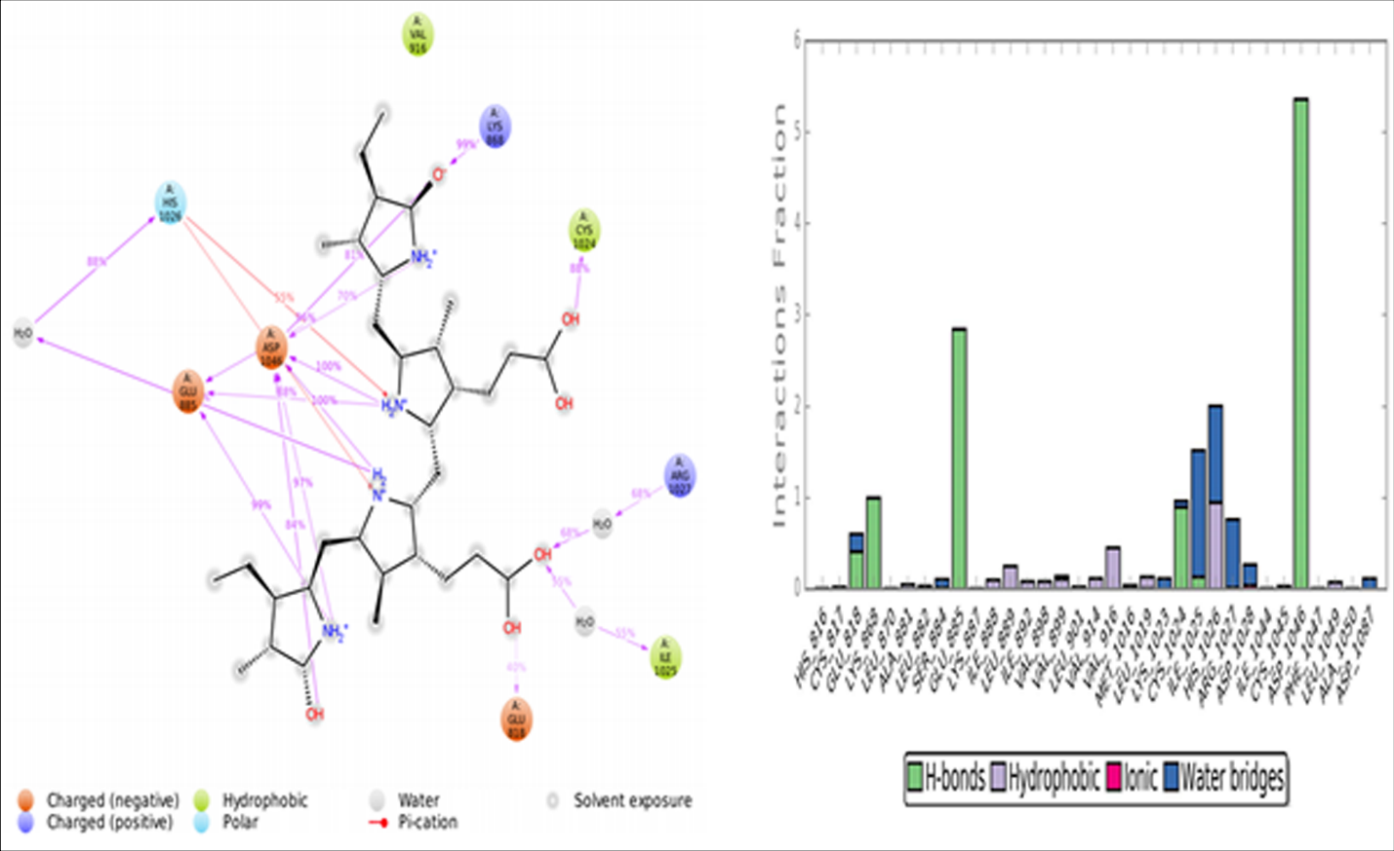
Protein-ligand interactions and contacts

**Figure 7:**
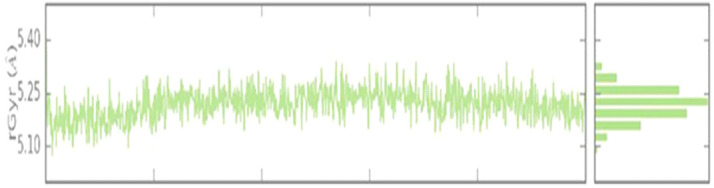
Radius of gyration (Rg) of VEGFR2-C-PC during the 100 ns MD simulation

One more crucial step is to analyze the H-bond interaction profiles of VEGFR2-C-PC docked complex. The H-bonds was key property in the computational drug design because of their strong binding influence on drug specificity, metabolism and adsorption. During the 100ns simulations the observed H-bond interactions VEGFR2 with C-PC were shown in Figure 5. The hydrogen bond interactions were observed throughout the 100 ns simulations period and it reveals that complex is relatively stronger and stable during the whole simulations period. Hydrogen bond interactions were formed with His816, Glu818, Lys868, Ser884, Glu885, Leu1019, Cys1024, Ile1025, Arg1027, Ile1044, Cys1045, Asp1046 and Phe1047 (Figure 5A). Whereas stable hydrophobic interactions were observed with Leu870, Ala881, Leu882, Ile888, Ile892, Val898, Val899, Leu901, Val914, Val916, Met1016, Leu1019, Cys1024, Leu1049 and Ala1050 (Figure 5B). Water mediated interactions were observed with His816, Cys817, Glu818, Ser884, Lys887, Asp889, Met1016, Lys1023, Cys1024, Ile1025, His1026, Arg1027, Asp1028, Ile1044, Cys1045, Asp1046 and Asp1087 (Figure 5C). Single ionic interaction was observed with Asp1028 and Pi-Pi interaction was observed with His1026 in 944 trajectories. The protein ligand contacts are of four different types namely Hydrogen bonds (H-bonds), hydrophobic, ionic and water bridges. The interaction of each sub types was analyzed using the 'Simulation Interaction Diagram' panel and the charts of stacked bars were standardized in the course time of the trajectory (Figure 6) VEGFR2-C-PC docking stability was also analyzed by the Rg analysis. The average Rg of the complex was observed at 1 Å (Figure 7), which indicates that binding of C-PC into the active site region of VEGFR2 does not induce any major conformational changes in the protein native structure.

## Conclusion

C-PC is reported as a potential angiogenesis inhibitor for VEGFR2 in combating cancer. C-PC showed H-bond interactions with residues on both sides of the Deusche Forschugsgemein-Schalt (DFG) loop (Asp1046-Phe1047-Gly1048). A hydrophobic association between the activation loop and the DFG residue (Gly1048) helps to inhibit the activity of VEGFR2 kinases.
